# Gut Microbiota-Derived Trimethylamine N-Oxide and NT-proBNP in Heart Failure: A Critical Review of Diagnostic and Prognostic Value

**DOI:** 10.3390/biomedicines14020287

**Published:** 2026-01-28

**Authors:** Natalia Anna Suchecka, Patrycja Popławska, Patrycja Obrycka, Agnieszka Frątczak, Ewa Tokarz, Julia Soczyńska, Sławomir Woźniak

**Affiliations:** 1 Student Scientific Group of Heart Diseases, Wroclaw Medical University, 50-556 Wroclaw, Poland; patrycja.poplawska@student.umw.edu.pl (P.P.); patrycja.obrycka@student.umw.edu.pl (P.O.); agnieszka.fratczak@student.umw.edu.pl (A.F.); ewa.tokarz@student.umw.edu.pl (E.T.); julia.niznik@student.umw.edu.pl (J.S.); 2 Division of Anatomy, Department of Human Morphology and Embryology, Faculty of Medicine, Wroclaw Medical University, 50-367 Wroclaw, Poland; slawomir.wozniak@umw.edu.pl

**Keywords:** heart failure, TMAO, trimethylamine N-oxide, gut microbiota, biomarkers

## Abstract

**Objective**: The study aims to evaluate the diagnostic and prognostic efficacy of gut-derived trimethylamine N-oxide (TMAO) as a molecular biomarker for heart failure (HF) in comparison to the N-terminal pro-B-type natriuretic peptide. **Background**: The clinical value of N-terminal pro-B-type natriuretic peptide (NT-proBNP) is frequently affected by non-cardiac physiological variables, including adiposity, advanced age, and renal clearance rates. Consequently, there is a compelling need for additional biomarkers. This analysis investigates TMAO as a critical mediator within the gut–heart axis, reflecting systemic inflammation and myocardial fibrosis secondary to intestinal dysbiosis. **Methods**: A comprehensive literature search was conducted using PubMed. Keywords such as “trimethylamine N-oxide”, “heart failure”, “heart failure with preserved ejection fraction” and “N-terminal pro-B-type natriuretic peptide” were used. The inclusion criteria comprised original research and literature reviews describing the pathophysiological mechanisms and clinical utility of TMAO in the context of HF diagnosis and prognosis. **Results**: The analyzed literature suggests that TMAO functions as an independent predictor of major adverse cardiovascular events, correlating with all-cause mortality and rehospitalization risk across all HF phenotypes. Furthermore, data indicate that using TMAO alongside NT-proBNP measurements may predict patient risk more accurately, particularly in patients where natriuretic peptide interpretation is traditionally obscured by comorbidities such as diabetes mellitus and chronic kidney disease. **Conclusions**: Although NT-proBNP remains the gold standard for acute diagnosis, TMAO provides significant value for long-term clinical management. By serving as a metabolic–inflammatory indicator, TMAO complements standard diagnostic panels, offering deeper insights into the prognostic trajectory and the underlying intestinal barrier integrity of patients with HF.

## 1. Introduction

Heart failure (HF) is a clinical syndrome that has assumed the proportions of a global pandemic [1]. Epidemiological data report a prevalence of 1–2% and 56 million affected individuals worldwide [2]. The disease predominantly affects regions with high levels of economic development and is more common in men [3]. Low- and middle-income countries are described as those whose inhabitants present with more advanced stages of HF and are often younger; in these settings the data indicate a higher proportion of affected women [4]. The incidence of the condition increases with age. HF is described as one of the principal causes of hospitalization among patients older than 65 years [3,5]. The disease imposes a burden not only on individuals but also on economies, being associated with recurrent episodes of prolonged inpatient care that generate substantial costs [5]. The literature lists, among contributors to HF, ischemic heart disease, arterial hypertension, and inflammatory and metabolic factors. The pathophysiology, however, is complex and encompasses mechanisms that initially serve compensatory roles—for example, the renin–angiotensin–aldosterone system and prolonged excessive activation of the sympathetic nervous system. An imbalance between the production and neutralization of ROS is also present, leading to endothelial dysfunction, inflammation, and cardiac remodeling [6]. In addition to the mechanisms already mentioned, HF is influenced by genetic and environmental factors, lifestyle, and comorbidities such as diabetes mellitus [7,8]. Insights into pathophysiological processes have enabled the identification of biomarkers. These are employed for diagnosis and monitoring of patients as well as for risk stratification [9]. Beyond the classical N-terminal pro-B-type Natriuretic Peptide (NT-proBNP), authors describe, in the context of HF, markers such as troponins, soluble ST2, and galectin-3, and they propose categorizations of markers linked to inflammation, oxidative stress, remodeling, hemodynamics, neurohormonal activity, and other pathways [10].

The aim of this work is a critical analysis of the diagnostic and prognostic value of TMAO in the context of HF as a complement to NT-proBNP, and a comparison of the two markers on the basis of the latest literature. We undertook an in-depth analysis of the topic, beginning with fundamentals such as the biosynthesis of TMAO and modulators of its concentration—for example, dietary influences, medications, lifestyle factors, and comorbid pathologies. We draw attention to mechanisms linking TMAO with HF, presenting pathways leading to inflammation, endothelial dysfunction, fibrosis, and remodeling. We emphasize the role of mitochondrial dysfunction and hormonal disturbances and identify the vicious circle associated with intestinal hypoperfusion and dysbiosis in HF. We summarize the latest available clinical evidence, analyzing the potential of TMAO as a prognostic, predictive, and diagnostic marker. The manuscript concludes with a discussion of challenges and limitations in the use of TMAO as a biomarker, identifying existing gaps in the literature, and proposing potential directions for future research that might enable TMAO to be employed in clinical practice.

## 2. Biological Basis and Clinical Significance

The natriuretic peptides remain the most commonly used and most readily available biomarkers in clinical practice for both acute and chronic HF [9]. NT-proBNP is often regarded as the gold standard for prognostic and risk assessment in HF [11]. Elevations in NT-proBNP correlate strongly with poorer clinical outcomes, and serial measurements are more useful for treatment prognostication than single determinations [12]. Authors note, however, that its interpretation should occur within a broader clinical context—for example, in the assessment of HF with preserved ejection fraction [11]. The literature indicates that NT-proBNP concentration may be influenced by determinants not directly related to cardiac function—for instance, obesity. Studies report that individuals with obesity exhibit lower concentrations of natriuretic peptides. Moreover, the study by Kozhuharov et al. confirmed that NT-proBNP provides greater diagnostic precision in non-obese patients than in obese patients [13]. Higher NT-proBNP concentrations are also observed in older individuals, which may create a more frequent need for further diagnostic evaluation in these patients and the potential risk of under investigation among younger patients [14]. We emphasize these limitations and regard them as important in light of evidence indicating a rising prevalence of obesity and an ageing population. It is projected that between 2010 and 2030 the number of obese adults will increase by more than 115%, reaching 1.13 billion [15]. Regarding the latter, the World Health Organization reports that the number of people aged 80 years and older is estimated to triple between 2020 and 2050, reaching 426 million [16]. Data also indicate that higher NT-proBNP concentrations are observed in women compared with men, and elevated values are additionally noted among those receiving hormonal therapy [11]. Important limitations are also associated with the renal clearance of NT-proBNP. This fact logically explains the existence of elevated marker concentrations in patients with impaired renal function. A similar tendency is observed in the context of anemia, although the explanation for this phenomenon is not yet fully elucidated. The literature notes that the application of NT-proBNP for, inter alia, estimation of cardiovascular events remains under discussion owing to the aforementioned factors [17]. Because of the mechanism of NT-proBNP generation, elevated values are also observed in atrial fibrillation (AF). Findings from the study by Jones et al. indicate that this parameter offers greater diagnostic precision for HF in patients without AF than in those with AF. The authors note that, in patients with AF, consideration of HF in diagnostic work-up might potentially be warranted only at higher NT-proBNP thresholds, while being mindful of the possibility of missed diagnoses [18].

In view of these limitations, we consider it necessary to pursue novel biomarkers that might potentially address the problems described or complement existing panels. Given that HF is a disease associated with inflammation and metabolic factors [6], it is reasonable to consider components that modulate these processes. Evidence exists for the role of gut microbiota in modulating cardiovascular risk. Authors indicate disrupted intestinal barrier integrity and the potential translocation of bacteria and metabolites that activate systemic inflammation. Among these are lipopolysaccharides, zonulin, short-chain fatty acids, bile acids, and TMAO [19]. We decided to focus on the latter. By way of introduction, the history of TMAO as a parameter associated with prediction of increased cardiovascular event risk dates back to 2013 [20]. TMAO is a metabolite produced by the gut microbiota from L-carnitine, choline, and betaine—nutrients found, respectively, in red meat, egg yolks, and certain seafood. Choline and carnitine are utilized in the production of trimethylamine (TMA), which is subsequently metabolized by hepatic enzymes to TMAO [21]. TMAO has been linked to processes that result in endothelial dysfunction and inflammation. It has the potential to affect lipid metabolism and platelet reactivity [22]. According to the most recent publications, there are suggestions that TMAO participates in the development and progression of HF. The literature indicates that its concentrations are elevated in patients with HF and that TMAO is being investigated as a prognostic marker in this disease [23].

## 3. Introduction to TMAO

TMAO is a biochemical molecule that is classified as an amine oxide. It is widely distributed in nature, as it can be found not only in animals, but also in plants or fungi [24]. Although numerous studies have demonstrated its link to adverse cardiovascular outcomes, TMAO also fulfills a critical biological role, acting as an osmolyte and protein stabilizer. In humans, its synthesis requires two consecutive steps: intestinal production of TMA by the gut microbiota, followed by liver oxidation to form the final metabolite [25].

### 3.1. Gut Microbiota and Sources of TMAO

Human gut microbiota comprises millions of microorganisms that are crucial in various processes, such as immunomodulation, preservation of the mucosal barrier’s structural integrity or host metabolic regulation [26]. It is also important to highlight their role in the biosynthesis of multiple chemical compounds, including TMAO.

Molecules, such as choline, phosphatidylcholine (lecithin), L-carnitine, betaine and ergothioneine predominantly found in animal-derived food act as substrates for intestinal TMA formation [27]. Main nutritional sources of choline and phosphatidylcholine include eggs, meat and dairy. Although choline can be synthesized endogenously, dietary intake is necessary to meet humans’ physiological demands. It is noteworthy that only excessive amounts of consumed choline (>27 mmol/L in adults) are converted into TMA, as lower quantities are normally absorbed in the small intestine and do not reach the large bowel [28,29]. Another significant source of TMA is L-carnitine, largely found in red meat and, like choline, mainly acquired exogenously. Likewise, higher intake of L-carnitine (0.5–6 g) exceeds its absorptive capacity and the compound becomes a substrate for gut microbiota [30]. It is also important to consider L-carnitine present in popular energy drinks, as their overconsumption may contribute to increased TMAO concentrations [31]. In contrast to the previously described molecules, betaine is more common in plants such as spinach and wheat, rather than in animal-based products [22]. Lastly, ergothioneine, which is abundant in certain mushroom species, can also be converted into TMA [30,32]. Since TMAO naturally occurs in certain marine animals, their ingestion directly increases TMAO level in the blood [28].

TMA-producing bacteria within the intestinal microbiota constitute a large taxonomically diverse group, including species from genera such as *Lactobacillus*, *Bacteroides*, *Fusobacterium* and *Escherichia*. Through the activity of key enzymes—betaine reductase, choline-TMA lyase, carnitine monooxygenase and ergothionase—these microorganisms convert suitable substrates into TMA. Then, the generated product is absorbed into the bloodstream and transported through portal circulation to the liver, where it is oxidized to form TMAO [33,34]. Given that antibiotics kill the bacteria responsible for TMA production, they may substantially reduce total TMAO levels. Therefore, TMAO cannot be considered as a reliable biomarker for cardiovascular disease (CVD) during ongoing antibiotic therapy [35].

In HF and similarly in inflammatory bowel disease, the composition of the intestinal microbiota undergoes significant alterations, characterized by an expansion of potentially pathogenic bacteria and a decline in beneficial species. The reduction in microbial diversity further worsens this imbalance. Notably, these changes may modulate circulating TMAO levels by influencing TMA synthesis in the large bowel. Moreover, the increased intestinal permeability observed in HF may facilitate the translocation of pathogenic microbes and their metabolites into systemic circulation, promoting chronic inflammation and contributing to the progression of HF [20,27,36,37].

### 3.2. TMAO Metabolism

Having reached the liver, TMA is oxidized to TMAO by flavin-containing monooxygenases, primarily flavin monooxygenase 3 *(FMO3)* [38]. Expression of *FMO3* gene begins within the first two years of life and continues to rise during childhood, reaching its peak in adulthood. This enzyme is responsible for the oxygenation of numerous compounds, mainly tertiary amines and sulfides. Interestingly, although *FMO3* is most abundant in hepatic cells, it can also be found in the skin, pancreas and adrenal glands [39].

The catalytic efficiency of *FMO3* may vary depending on the genetic polymorphisms, as many variants have been identified so far. While some mutations do not appear to affect *FMO3* enzymatic activity, others alter its structure and function, leading to impaired TMAO production and limiting its use as a biomarker. Such a situation occurs in trimethylaminuria, a recessively inherited disease, also known as ‘fish odor syndrome’. The majority of the recognized gene variants in trimethylaminuria are missense mutations, which variably decrease *FMO3* catalytic performance, and in some cases, abolish TMAO synthesis entirely. Moreover, studies have shown that three of the most prevalent *FMO3* gene polymorphisms in the general population (*V257M*, *E308G* and *E158K*) presented reduced activity in comparison to the wild type *FMO3*, which should also be considered when using TMAO as a molecular marker [40,41,42].

In addition to those previously mentioned, there are many other factors influencing the expression of *FMO3* gene and therefore affecting plasma TMAO levels. For example, hepatic insulin resistance has been shown to enhance its activity [43]. Similarly, age-related changes correlate with *FMO3* upregulation in adipocytes [44]. Furthermore, studies in mice reveal a significant difference between male and female individuals, associated with testosterone suppressing *FMO3* expression and estrogen stimulating it [45]. In humans, however, such a gender distinction is much less evident or even absent [46].

### 3.3. Modulators of TMAO Concentration

The role of diet and antibiotic treatment in modulating TMAO circulation has already been addressed in this article. Supplementation with probiotics and prebiotics (e.g., *Lactobacillus plantarum* GLP3), through their beneficial impact on the gut microbial composition, may decrease TMA and TMAO levels [47,48]. What is more, certain drugs, including metformin and aspirin, can reduce the formation of these metabolites by limiting TMA-lyase activity [49,50]. Berberine is another compound with the potential to decrease circulating TMAO in blood [51]. It is also important to emphasize the role of physical activity, which, alongside the reduction in dietary precursors, may serve as a lifestyle intervention that supports lowering TMAO levels [52].

Elevated TMAO concentrations have been associated with metabolic disorders such as insulin resistance, impaired fasting glucose, and ultimately, in type 2 diabetes (T2D) [24]. A similar relationship has been reported in patients with non-alcoholic fatty liver disease [53]. In contrast to NT-proBNP, TMAO as a marker does not exhibit the obesity paradox [54]. Conducted studies have shown that variables such as BMI, insulin resistance and lipid profiles positively correlate with TMAO levels, although only age has been identified as statistically significant factor. Furthermore, potential genetic contributions to elevated TMAO levels have been suggested, as higher amounts of this compound have been linked to a family history of obesity and diabetes [55]. Importantly, these CVD risk factors, together with hypertension, atherosclerosis and cigarette smoking, may independently exacerbate gut dysbiosis, further supporting the expanding role of the gut microbiome in CVD pathophysiology [55,56].

Excretion through the kidneys, predominantly via glomerular filtration, is the major mechanism for TMAO removal, as non-renal pathways contribute only minimally [57]. Consequently, circulating TMAO concentrations are elevated in chronic kidney disease (CKD), reflecting reduced renal clearance efficiency [58]. Recent studies in CKD population suggest that TMAO may serve diagnostic value in differentiating CKD patients from healthy individuals, and higher TMAO levels have been associated with a greater risk of CKD development [59]. Given the bidirectional relationship between CKD and HF, the onset of one condition promotes initiation of the other [60]. Similarly to findings in CKD, elevated TMAO concentrations are linked to a higher risk of HF incidence (rising by up to 15% per doubling) and may serve as a poor prognostic marker, with increased circulating TMAO correlating with a greater likelihood of major adverse cardiovascular events (MACEs) [21,61].

## 4. TMAO Contribution to HF

**Figure 1:**
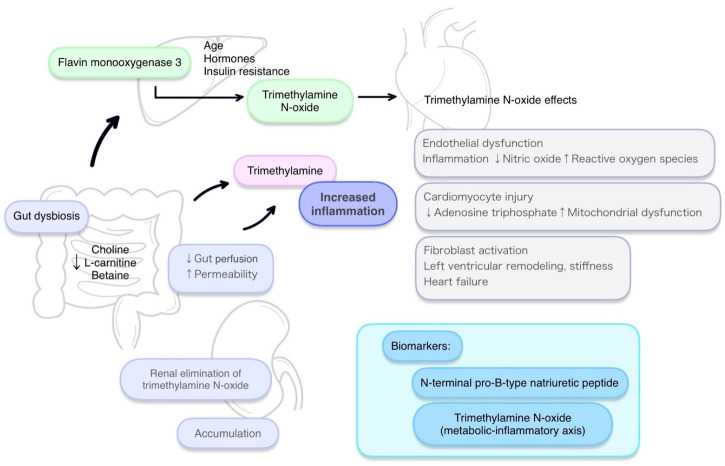
The gut–heart axis: TMAO metabolism and its impact on cardiovascular pathology.

TMAO has become, in recent years, one of the most intensively studied metabolites associated with CVD, including HF. A growing body of data indicates that TMAO is not merely a biomarker of microbial dysbiosis or unfavorable dietary habits, but an active pathogenic factor that modulates the function of vessels, cardiomyocytes, kidneys, and the neurohormonal system [62]. These mechanisms form a complex cascade that drives cardiac remodeling, systolic and diastolic dysfunction, and the progression of HF symptoms (Figure 1). While numerous existing reviews have focused primarily on the association between TMAO and clinical outcomes—such as mortality and hospitalization—recent advances now allow for a more integrated, mechanistic interpretation that links TMAO not only to prognosis, but also to specific myocardial, vascular, and neurohormonal alterations underlying HF progression, thereby providing a rationale for phenotype-specific diagnostic and therapeutic assessment. By incorporating vascular, myocardial, and neurohormonal markers that extend beyond traditional clinical endpoints, this approach is particularly relevant for identifying specific HF phenotypes, characterizing gut barrier dysfunction, and evaluating TMAO as a potential tool for therapeutic monitoring. The current state of knowledge on the multiorgan effects of TMAO and its role in the pathophysiology of HF is presented below [63,64].

TMAO is formed through a multistep metabolic process in which the interaction between the gut microbiota and the liver plays a key role. Gut bacteria, especially those belonging to the phyla *Firmicutes* and *Proteobacteria*, metabolize choline, carnitine, and betaine—compounds abundantly present in the Western diet—into TMA [65]. TMA is then transported to the liver, where it is oxidized by *FMO3* into TMAO. *FMO3* activity is modulated by genetic, inflammatory, hormonal, and dietary factors, which partially explains interindividual variability in TMAO levels [66].

In HF patients, significant disturbances in the composition of the gut microbiota (so-called dysbiosis) are observed, including reduced diversity, dominance of pro-inflammatory bacteria such as *Bacteroides*, *Prevotella*, *Enterococcus*, and *Enterococcaceae*, as well as impaired integrity of the intestinal barrier. These phenomena promote TMA overproduction and elevated circulating TMAO concentrations. Concurrent renal failure further limits its excretion, creating conditions for additional metabolite accumulation and persistence of adverse metabolic effects [67,68].

One of the best—characterized effects of TMAO, central to HF pathogenesis, is the induction of endothelial dysfunction. TMAO reduces nitric oxide bioavailability by increasing reactive oxygen species (ROS) production and impairing nitric oxide synthase activity via competition with L-arginine for the catalytic site of the enzyme. This results in impaired vasodilation, increased vascular tone, and microangiopathy limiting myocardial perfusion [69].

At the molecular level, TMAO activates the NF-κB pathway, leading to increased expression of pro-inflammatory cytokines (IL-6, TNF-α, IL-1β) and adhesion molecules (ICAM-1, VCAM-1). This leads to enhanced leukocyte adhesion, inflammation of the vascular wall and accelerated atherogenesis [25,70]. TMAO also increases platelet reactivity by enhancing Ca^2+^ release, thereby promoting microthrombi formation, which can further exacerbate myocardial ischemia and functional deterioration [71].

TMAO also acts directly on cardiomyocytes, disrupting mitochondrial function, increasing ROS production and weakening antioxidant defense mechanisms, including reduction in the activity of enzymes such as SIRT1—a deacetylase from the sirtuin family that post-translationally regulates cellular processes including inflammation and oxidative stress—leading to cell injury [72,73]. Furthermore, it has been shown that TMAO decreases phosphocreatine and reduces ATP levels by downregulating the respiratory chain complex IV, impairing mitochondrial oxidative phosphorylation and limiting cardiomyocyte energy production [74].

TMAO also induces death of endothelial cells through pyroptosis by activating the NLRP3 inflammasome, caspase-1, and promoting the formation of membrane pores via Gasdermin D. This process is linked to increased ROS and inhibition of mitochondrial aldehyde dehydrogenase 2, which increases cell susceptibility to death and may contribute to vascular dysfunction [75].

The most characteristic consequence of TMAO action is stimulation of cardiac fibroblasts and promotion of fibrosis. This occurs mainly via activation of the TGF-β1/Smad3 axis, promoting expression of collagen types I and III and alpha-smooth muscle actin. Fibrosis increases myocardial stiffness, impairs diastolic function and promotes conduction disturbances, creating an arrhythmogenic substrate [76,77]. These mechanisms may be related to acetylation of C/EBPβ, which in in vitro models regulates alpha-smooth muscle actin promoter activity and is critical for epithelial-to-mesenchymal transition and overproduction of collagen type I, suggesting similar epigenetic processes may participate in TMAO-induced cardiac fibrosis [78]. Importantly, this highlights mechanistic myocardial alterations extending beyond conventional measures such as left ventricular ejection fraction (LVEF). In the analyzed cohort of post ST-elevation myocardial infarction patients, it was demonstrated that rising TMAO concentration during the first 3 months after percutaneous coronary intervention significantly correlated with worse systolic and diastolic left ventricular (LV) function after 12 months. Individuals who developed group 4 final left ventricular remodeling or ≥grade II left ventricular diastolic dysfunction had higher levels of TMAO, brain natriuretic peptide (BNP), and high-sensitivity C-reactive protein (CRP), highlighting the role of these biomarkers in adverse cardiac remodeling progression [79].

Additionally, TMAO increases aortic stiffness both in vivo and ex vivo by enhancing oxidative stress and collagen crosslinking via advanced glycation end-products. These effects were observed at plasma TMAO concentrations of ~30 µM, typical for mouse models receiving a TMAO-supplemented diet, corresponding to values observed in humans at elevated cardiovascular risk [80]. Aortic stiffness may therefore represent an additional mechanistic marker linking TMAO to vascular and ventricular uncoupling in HF.

TMAO may also disrupt cholesterol metabolism by inhibiting bile acid synthesis pathways, including the CYP7A1 and CYP27A1 enzymes, thereby limiting cholesterol excretion. Consequently, impaired reverse cholesterol transport promotes cholesterol retention in the liver and vascular wall, further increasing cardiovascular risk [65].

Another important aspect of TMAO-mediated HF progression is increased vascular responsiveness to angiotensin II, leading to enhanced vasoconstriction and elevated arterial pressure. This mechanism involves increased angiotensin II sensitivity via angiotensin II type 1 receptor activation and ROS overproduction, promoting sodium and water retention and potentially aggravating cardiac remodeling [81].

At the level of the sympathetic nervous system, TMAO increases neuronal activity in the cardiac stellate ganglion and in the paraventricular nucleus of the hypothalamus, leading to enhanced adrenergic tone and sympathetic overactivity. These effects contribute to the shortening of ventricular refractory period, promote ventricular arrhythmias, and worsen LV function, especially in advanced HF [82].

It is also important to highlight the vicious cycle within the gut–heart axis. In HF, impaired perfusion and intestinal congestion lead to damage to the epithelial barrier and dysbiosis, which enhances bacterial TMA production and further increases circulating TMAO concentration. Elevated TMAO, associated with poor prognosis, accelerated myocardial remodeling and progression of HF, additionally worsens cardiac function, sustaining the self-perpetuating pathomechanism [83].

In conclusion, TMAO contributes multidirectionally to HF progression by exacerbating endothelial dysfunction, oxidative stress, fibrosis, and disturbances in cardiomyocyte and neurohormonal system function. Higher TMAO concentrations correlate with adverse LV remodeling (advanced left ventricular remodeling, left ventricular diastolic dysfunction), increased aortic stiffness and elevated arterial pressure. These effects may differ across HF phenotypes, including HF with reduced ejection fraction (HFrEF), HF with mildly reduced ejection fraction (HFmrEF) and heart failure with preserved ejection fraction (HFpEF), as these entities are characterized by distinct patterns of ventricular remodeling, myocardial stiffness, vascular function and neurohormonal activation. This suggests potential phenotype-specific diagnostic and therapeutic implications of TMAO assessment, particularly in the context of vascular dysfunction, myocardial fibrosis and ventricular–vascular coupling [84]. Meanwhile, HF aggravates intestinal dysbiosis and TMAO production, creating a self-perpetuating vicious circle, emphasizing the importance of targeted therapeutic strategies modulating gut microbiota and the TMA/TMAO metabolic pathway as potential tools for risk stratification and therapeutic monitoring in selected HF populations.

## 5. Clinical Evidence

Over the past decade, numerous clinical studies have investigated the potential role of TMAO as a biomarker in HF (Table 1). This section provides a comprehensive review of original research evaluating the prognostic, diagnostic, and predictive value of plasma TMAO levels in different HF populations.

### 5.1. TMAO and NT-proBNP

Several clinical studies have demonstrated that combining plasma TMAO measurements with NT-proBNP significantly enhances the prediction of adverse outcomes in patients with HF. In a prospective cohort study involving 189 IHF patients with LVEF below 45%, elevated levels of both TMAO and NT-proBNP were associated with the highest risk of all-cause mortality. Additionally, higher levels of both biomarkers were significantly correlated with longer hospitalization duration [85]. Supporting these findings, another study conducted in a northern Chinese cohort of 112 control participants and 184 HF patients identified TMAO as an independent predictor of HF. Plasma TMAO levels were significantly higher in HF patients and progressively increased with worsening NYHA class. Although TMAO correlated positively with NT-proBNP, its diagnostic accuracy was lower than that of NT-proBNP [86]. Similarly, in HFpEF patients, TMAO levels were significantly elevated compared with controls. ROC analysis showed good predictive value for TMAO and NT-proBNP, with their combination further improving prediction. Multivariate analysis confirmed TMAO as an independent risk factor for HFpEF [87]. Furthermore, in patients with chronic systolic HF, elevated plasma levels of TMAO, along with its dietary precursors choline and betaine, were associated with more advanced diastolic dysfunction and higher NT-proBNP levels. While all three metabolites predicted worse 5-year clinical outcomes, only TMAO remained an independent prognostic marker after adjustment for cardiorenal variables, emphasizing its value in long-term risk stratification in systolic HF [88]. A similar conclusion was drawn in a study involving 720 patients with stable HF. It concluded that elevated TMAO was modestly correlated with BNP and independently predicted a 3.4-fold increased 5-year mortality risk after adjusting for traditional risk factors and BNP levels, highlighting its prognostic value in HF [89]. Overall, these studies consistently demonstrate that elevated plasma TMAO levels are independently associated with adverse outcomes in HF and, when combined with NT-proBNP, enhance risk stratification beyond traditional biomarkers.

### 5.2. Diabetes

Detecting HF in patients with T2D remains particularly challenging, as insulin resistance, obesity, and chronic inflammation can reduce the diagnostic utility of traditional biomarkers [90]. In this context, circulating TMAO has emerged as a potential candidate for improving cardiovascular risk assessment in diabetic populations. However, current evidence remains inconsistent. In a prospective study that followed 1468 T2D patients over a median of 7.3 years, elevated baseline levels of TMAO and its precursors were initially linked to a higher risk of HF-related hospitalization. Yet, after adjustment for conventional cardiac and renal risk factors, these associations lost statistical significance, and only homocysteine remained an independent predictor of all-cause mortality [91]. These findings suggest limited prognostic value of TMAO as a standalone marker of HF in T2D. In contrast, data from another cohort involving 595 T2D patients with a median follow-up of 10 years demonstrated that higher plasma TMAO levels were independently associated with an increased risk of cardiovascular mortality, even after extensive adjustment for clinical and biochemical confounders, including branched-chain amino acids (BCAAs). Notably, while BCAAs showed no association with cardiovascular mortality, TMAO remained a strong independent predictor [92]. Overall, these studies suggest that while TMAO shows limited value for predicting HF hospitalizations in T2D patients, it may serve as an independent marker of long-term cardiovascular mortality in this population.

Beyond T2D, similar associations have been reported in type 1 diabetes. In a large cohort of 1159 individuals with type 1 diabetes, elevated baseline plasma TMAO concentrations were associated with an increased risk of all-cause and cardiovascular mortality, CVD events, and progression to end-stage renal disease over a median follow-up of 15 years. These associations remained significant after adjustment for conventional cardiovascular risk factors, but lost significance after adjusting for baseline estimated glomerular filtration rate (eGFR), suggesting that TMAO may primarily reflect impaired renal function in this population [93].

Taken together, current evidence indicates that while elevated TMAO levels in diabetes are associated with adverse cardiovascular outcomes, their prognostic significance largely depends on renal function.

### 5.3. CKD

Given that TMAO is predominantly cleared by the kidneys, its circulating levels rise in parallel with declining renal function, making CKD a critical clinical context for interpreting its prognostic relevance [94]. In a prospective cohort study of CKD stage 3–5 patients, plasma TMAO concentrations were significantly higher in individuals with established CKD compared with individuals without kidney impairment and were associated with increased risk of all-cause mortality during 5-year follow-up. This relationship remained significant after adjustment for traditional cardiovascular risk factors. TMAO levels correlated positively with inflammatory markers, including IL-6, fibrinogen, and high-sensitivity CRP. Moreover, plasma TMAO concentrations normalized after kidney transplantation but were not reduced by dialysis, indicating that functional renal clearance rather than extracorporeal removal determines TMAO burden in advanced CKD. Beyond mortality risk, accumulating evidence indicates that TMAO may reflect systemic processes linked to CKD progression [95].

Consistent findings were reported in a population-based cohort of 5469 participants, where higher TMAO levels were associated with increased all-cause mortality. Notably, this association was evident only in individuals with eGFR < 90 mL/min/1.73 m^2^ and lost significance after adjustment for albuminuria and eGFR, suggesting that impaired renal function may partly mediate the relationship between TMAO and adverse outcomes [96].

Collectively, current evidence indicates that elevated TMAO levels in CKD are closely associated with increased mortality, systemic inflammation, and disease progression, yet their prognostic interpretation remains challenging due to the strong dependence of circulating TMAO on renal function and reduced clearance capacity

### 5.4. Peripheral Artery Disease (PAD)

PAD is a chronic atherosclerotic condition in which TMAO may offer valuable prognostic information by improving cardiovascular risk assessment. In an initial study, higher fasting plasma TMAO levels were associated with an increased risk of MACEs and all-cause mortality over a 5-year follow-up in patients with PAD. This relationship remained significant after adjustment for traditional cardiovascular risk factors, supporting the independent predictive value of TMAO in PAD populations [97].

Subsequent analysis in a cohort of 262 patients confirmed these findings. Notably, elevated TMAO (>2.26 µmol/L) remained an independent predictor after adjustment for conventional risk factors, inflammatory biomarkers, and the presence of coronary artery disease. TMAO significantly improved risk stratification metrics such as the net reclassification index and the area under the receiver operating characteristic curve [98]. In addition to its association with adverse outcomes, TMAO has been shown to correlate with disease severity. In 262 symptomatic PAD patients categorized as intermittent claudication or critical limb ischemia, TMAO levels were significantly higher in critical limb ischemia compared with intermittent claudication. Patients with TMAO above 2.26 µmol/L exhibited a greater risk of cardiovascular death, independent of conventional risk factors [99]. These findings highlight that TMAO not only identifies PAD patients at high risk of mortality but also reflects the severity of peripheral arterial disease.

Overall, these studies demonstrate that elevated plasma TMAO serves as an independent marker of disease severity and adverse cardiovascular risk in PAD, offering potential value for improved risk stratification and clinical management.

### 5.5. Acute Heart Failure (AHF)

AHF remains a clinical condition with high morbidity and mortality, where early risk stratification is essential. Plasma TMAO, a gut microbial-derived metabolite, has recently emerged as a potential prognostic biomarker in this setting [61]. In a prospective study involving 972 AHF patients, elevated plasma TMAO levels were associated with increased risk of in-hospital mortality, one-year all-cause mortality, and rehospitalization. Notably, when combined with NT-proBNP, TMAO further improved risk stratification, particularly in identifying patients at the highest risk of adverse outcomes. However, the prognostic utility of TMAO was attenuated after adjustment for renal function markers, highlighting the influence of kidney function on TMAO interpretation [100]. These findings suggest that TMAO provides valuable prognostic information in AHF, especially when considered alongside established biomarkers such as NT-proBNP although its clinical utility may be optimized by accounting for renal function.

### 5.6. Coronary Artery Disease

Growing evidence indicates that TMAO also possesses prognostic value in patients with stable coronary artery disease. In a large prospective cohort including 2235 individuals undergoing elective coronary angiography, higher fasting plasma TMAO levels were associated with a nearly fourfold increase in 5-year all-cause mortality. Importantly, this relationship remained significant after adjustment for traditional cardiovascular risk factors, systemic inflammation (high-sensitivity CRP), and renal function (eGFR). Furthermore, TMAO provided incremental prognostic value when added to established risk models, improving risk reclassification and long-term mortality prediction in stable coronary artery disease populations [101].

Similar observations were reported in patients with suspected functionally relevant coronary artery disease (fCAD). In a prospective study, plasma concentrations of TMAO and its dietary precursors—betaine, choline, and carnitine—were significantly elevated in fCAD patients. Although their diagnostic performance for detecting functionally relevant stenosis was limited, TMAO remained a strong independent predictor of both all-cause and cardiovascular mortality over 5 years of follow-up, even after adjusting for renal function and other confounders [102]. These results suggest that while TMAO has limited utility as a diagnostic biomarker in fCAD, it offers substantial prognostic information regarding long-term outcomes.

Collectively, these studies indicate that while TMAO has limited diagnostic value in coronary artery disease, elevated plasma levels consistently predict adverse long-term outcomes and enhance prognostic risk stratification in chronic coronary syndromes. However, its prognostic value should be interpreted with caution due to the potential confounding by renal function and heterogeneity across study populations, and further standardized, phenotype-specific investigations are warranted.

## 6. Discussion

According to the available clinical evidence, patients with HF and other CVDs consistently exhibit elevated TMAO levels, which remain positively correlated with cardiovascular events [103,104]. The most consistent data were obtained in relation to the prognostic value of TMAO in all-cause mortality and hospitalization risk [89,101,105]. However, some investigations suggest that, when classical risk factors and eGFR are considered in certain cohorts, the relationship between TMAO and hospitalization risk in HF loses its statistical significance after adjustment, implying that TMAO may not be an independent prognostic marker [91]. These findings indicate that the prognostic utility of TMAO may be limited to specific clinical contexts rather than being universally applicable.

Several clinical studies proved that the combination of TMAO and NT-proBNP improves overall risk stratification compared to the use of only one of those markers at a time, suggesting TMAO’s strong value as a complementary metabolic–inflammatory indicator alongside widely and predominantly used NT-proBNP [85,100,106]. The pathophysiological potential causative connection of TMAO to HF distinguishes it from the hemodynamic-effective NT-proBNP [107]; nevertheless, existing clinical data demonstrate only a correlation between TMAO and clinical outcomes in patients with HF [101,105]. Further studies investigating potential interventions that alter serum TMAO levels and their influence on HF are needed. The key question of whether lowering TMAO improves prognosis in CVD (particularly in HF) remains unanswered, as meta-analyses and randomized controlled trials (RCTs) on this topic do not currently exist.

Moreover, studies have shown that the correlation between elevated TMAO levels and HF may not appear in all ethnicities, particularly in Japanese cohorts, which exhibit higher levels than Caucasian and South Asian cohorts with HF but similar clinical outcomes [108]. Further meta-analyses are required to determine whether this difference results from different lifestyle factors and dietary modifications or specific genetic mutations, especially in FMO3 metabolism [109]. Dietary trials modulating TMAO levels have been conducted almost exclusively in healthy volunteers and have focused on metabolomic outcomes without assessing cardiac biomarkers, vascular function, or inflammatory markers [110,111]. This significant gap in research means that current dietary intervention strategies to modulate TMAO levels in the context of HF, and their impact on clinically relevant cardiac endpoints, remain largely unexplored. Overall, these findings suggest that the biomarker value of TMAO may vary across populations and may not apply equally to all patient groups.

Despite consistent results in many cohorts, the clinical applicability of TMAO remains limited, as the strength of its association with clinical endpoints varies substantially across comorbidities. Available data regarding patients with diabetes and HF remain inconsistent [91,112]. The inconsistency in patients with diabetes and HF, for instance, may stem from varying glycaemic control, renal function, or medication use across studies, highlighting the need for more standardized research designs. A significant limitation is the lack of randomized clinical trials and meta-analyses covering the use of TMAO as a prognostic or predictive biomarker in these specific cohorts.

Furthermore, TMAO, like NT-proBNP, is cleared by the kidneys [94,113], which creates interpretative confusion as to whether its elevated levels truly correlate as a prognostic biomarker in HF or are simply a result of decreased GFR. Randomized clinical trials or meta-analyses testing the utility of TMAO as a prognostic biomarker in patients with HF and CKD, especially in comparison to cohorts with only one of these conditions, will need to be conducted in the future. Multiple studies have shown that the correlation between TMAO levels and adverse cardiac outcomes and all-cause mortality lost statistical significance after adjustment for eGFR [96,114]. This suggests that the prognostic value of TMAO may be limited in patients with renal dysfunction, particularly as elevated TMAO may reflect impaired renal function more than gut-derived pathology contributing to the pathomechanism of HF.

Future studies could employ advanced statistical methods or use renal tubular function markers alongside eGFR to more accurately determine the independent contribution of TMAO to HF prognosis.

Most studies examining the association between TMAO levels and HF do not distinguish between cohorts with HF with reduced ejection fraction, mildly reduced ejection fraction, and HFpEF, but instead focus on its predictive or prognostic value in HF patients generally. Therefore, further RCTs and meta-analyses are needed to clarify the TMAO levels in these groups separately. Similarly, more studies are required to determine the prognostic value of TMAO in acute and chronic HF separately, although available data indicate that in both of those phenotypes, TMAO correlates with MACE [115,116].

Notably, patients with HFpEF exhibit higher levels of TMAO [87,106,117], supporting its proposed utility where NT-proBNP concentrations may be inconclusive. In patients with HFpEF, TMAO consistently appears as an independent risk factor for clinical exacerbation [117], but most studies have small cohorts and cross-sectional designs, which limits their statistical significance and generalizability. Therefore, larger, prospective, and longitudinal analyses are essential to validate the utility of TMAO in HFpEF and to establish its robust prognostic value.

Most researchers did not standardize TMAO assays with respect to diet, comorbidities, or medications, which potentially limits their use as an independent prognostic biomarker. This lack of standardization introduces significant variability and potential confounding, making it difficult to compare findings across studies and to establish the independent prognostic value of TMAO, as dietary intake or medication could directly influence circulating levels. Additionally, certain dietary precursors, such as choline or L-carnitine, may interfere with the objective assessment of TMAO levels when prognosticating MACE in HF [118,119]. Moreover, patients with a wide range of gastroenterological disorders, such as inflammatory bowel diseases, may be particularly difficult to assess in the context of a disrupted microbiome, resulting in fluctuating TMAO levels [120]. Altogether, these limitations suggest that TMAO’s utility may be restricted to carefully defined patient subgroups with standardized assessment conditions.

Most analyses determining the association between TMAO and negative cardiac outcomes failed to include mechanistic cardiac outcomes, except for LVEF and NT pro-BNP, particularly markers of myocardial fibrosis or oxidative stress, even though some studies measured hsCRP [61] or cardiac remodeling [79] in relation to TMAO and HF. More detailed studies could help establish a direct link between MACE and TMAO. Incorporating these mechanistic markers is essential to establishing a direct association between TMAO and MACE and to elucidating the specific pathways through which TMAO contributes to cardiac pathology.

Prospective studies confirming the prognostic value of TMAO have relied solely on clinical endpoints and single baseline measurements with minimal assessment of intermediate metabolic mediators [89,104]. Importantly, these cohorts lacked serial measurements of TMAO, NT-proBNP, eGFR, or any remodelling biomarkers, preventing evaluation of temporal relationships and biological plausibility. Consequently, it remains unclear whether TMAO reflects underlying risk factors such as CKD, dietary modification, and diabetes, or acts as a mediator of HF progression. Therefore, TMAO may currently serve best as a complementary biomarker, with its independent prognostic value likely limited to select patient populations or subclinical disease.

Despite substantial and consistent clinical evidence linking TMAO to increased risk of mortality and hospitalization in patients with HF, this biomarker does not meet the necessary criteria to be included in HF management guidelines, primarily due to a lack of interventional evidence. No RCTs in patients with HF have shown that lowering TMAO levels improves clinical outcomes or that TMAO-guided management alters therapy or prognosis.

Currently, TMAO is not a therapeutic target, but merely a statistically correlated HF biomarker.

To establish TMAO as a reliable prognostic biomarker in HF, large, well-phenotyped cohorts and longitudinal interventional studies with repeated measurements modulating TMAO levels are essential to define causality and determine whether TMAO could be useful in reclassifying HF patients into different treatment pathways or whether dietary interventions based on TMAO levels would improve clinical outcomes.

## 7. Conclusions

Various studies in recent years have shown that TMAO strongly correlates with overall mortality and risk of hospitalization in patients with all HF phenotypes. In contrast to NT-proBNP, TMAO reflects metabolic wall stress and inflammatory changes associated with gut microbiota alterations rather than hemodynamic abnormalities, highlighting its auxiliary yet independent role as a potential prognostic biomarker in HF. However, none of the studies confirmed the superiority of TMAO over NT-proBNP; therefore, it is concluded that NT-proBNP remains the primary prognostic biomarker in HF, limiting the role of TMAO to an additional supportive parameter in patients for whom NT-proBNP results are inconclusive, such as those with HFpEF or HF and diabetes, CKD, or obesity. When combined with NT-proBNP, TMAO improves risk stratification, particularly in predicting long-term mortality and hospitalization risk, which may be useful for early identification of high-risk patients across various clinical settings. Further research should be conducted to establish standardized cutoff points as prognostic markers and to define specific indications for determining TMAO levels. Given the gut-derived origin of TMAO, there is a strong need for studies to determine whether dietary modifications that alter the gut microbiota can lower TMAO levels and subsequently improve the prognosis of patients with HF. Despite its promising role, a fundamental challenge remains in definitively establishing TMAO as a causal mediator of HF progression rather than a reflection of underlying risk factors, which necessitates rigorous longitudinal interventional studies.

## Figures and Tables

**Table 1:** Clinical evidence for trimethylamine N-oxide as a biomarker in heart failure.

Author, Year	Type of Study	Sample Size	Inclusion Criteria	Measurements	Results
Qiu W., 2022 [85]	Prospective cohort study	189 patients	Patients with left ventricular ejection fraction (LVEF) < 45% due to coronary artery disease, hospitalized between March 2016 and December 2020	Measurement of baseline plasma trimethylamine N-oxide (TMAO) and N-terminal pro-B-type natriuretic peptide (NT-proBNP levels)	Elevated TMAO combined with NT-proBNP improved all-cause mortality prediction, with the highest risk observed in patients with dual biomarker elevation
Dong Z., 2020 [86]	Observational study	296 patients (184 heart failure (HF) patients, 112 control group)	Diagnosed HF patients and non-HF controls, both from northern Chinese population	Measurement of plasma TMAO and NT-proBNP levels	Plasma TMAO levels were significantly higher in HF patients and increased progressively with NYHA class. TMAO was an independent predictor of HF and correlated positively with NT-proBNP, although its diagnostic performance was lower than NT-proBNP
Dong Z, 2021 [87]	Observational study	118 patients (61 heart failure with preserved ejection fraction (HFpEF) patients, 57 control group)	HF with preserved ejection fraction, healthy controls	Measurement of plasma TMAO and NT-proBNP levels with biochemical examination of all patients	Plasma TMAO levels were significantly higher in HFpEF patients than in controls. TMAO was an independent risk factor for HFpEF and correlated with NT-proBNP and renal function markers, with lower diagnostic performance than NT-proBNP
Tang W.H.W., 2015 [88]	Observational cohort study	112 HF patients	Patients with chronic systolic HF with comprehensive echocardiographic evaluation	Measurement of plasma TMAO, choline, betaine levels and echocardiographic evaluation	TMAO, choline, and betaine were linked to worse diastolic dysfunction and higher NT-proBNP, but only elevated TMAO predicted poor prognosis after cardiorenal adjustment
Tang W.H.W., 2014 [89]	Prospective cohort study	720 patients	Patients with stable HF	Measurement of fasting plasma TMAO, Brain Natriuretic Peptide (BNP) and 5-year follow-up	Elevated TMAO independently predicted 5-year mortality, even after adjusting for traditional risk factors and cardiorenal index
Baldassarre S., 2017 [90]	Population-based study	3244 patients (1364 with diabetes, 1880 without)	Adults aged 45–74 years without HF, with and without diabetes	Measurement of NT-proBNP and BMI	NT-proBNP was lower in overweight/obese individuals, partly due to insulin resistance and inflammation, while diabetes increased the odds of high NT-proBNP
Wargny M., 2022 [91]	Prospective cohort study	1468 patients (data available for 1349)	Type 2 diabetes (T2D)	Measurement of plasma TMAO, precursors and thio-amino acids	TMAO and its precursors were not independently associated with HF hospitalization or cardiovascular events, only homocysteine predicted all-cause mortality
Flores-Guerrero J.L., 2021 [92]	Prospective cohort study	595 patients	T2D	Measurement of plasma TMAO and branched-chain amino acids (BCAAs), 10-year follow-up for cardiovascular mortality	Higher plasma TMAO, but not BCAAs, independently predicted increased cardiovascular mortality in T2D patients
Winther S.A., 2019 [93]	Prospective cohort study	1159 patients	Type 1 diabetes	Measurement of plasma TMAO, follow-up for cardiovascular and renal outcome	Higher TMAO was initially associated with mortality, CVD (cardiovascular disease), and renal events, but associations were no longer significant after adjusting for baseline estimated glomerular filtration rate (eGFR)
Pelletier C.C., 2019 [94]	Prospective cohort study	124 patients	Patients with chronic kidney disease (CKD) and control group	Measurement of plasma TMAO, Trimethylamine (TMA), choline, betaine, carnitine and assessment of renal clearance and GFR	Plasma TMAO inversely correlated with GFR, with renal clearance largely via glomerular filtration and accumulation in CKD driven by reduced filtration, not tubular handling, and effectively removed by hemodiafiltration
Missailidis C., 2016 [95]	Prospective observational study	259 patients (179 with CKD, 80 in control group)	Patients with CKD stages 3–5 and control group	Measurement of plasma TMAO, choline, betaine, inflammatory markers and follow-up for all-cause mortality	TMAO strongly correlated with renal function and inflammation; levels normalized after renal transplantation and independently predicted all-cause mortality in CKD 3–5 patients
Gruppen E.G., 2017 [96]	Prospective cohort study	5469 patients (322 died)	General population	Measurement of plasma TMAO and median 8.3-year follow-up for all-cause mortality	Higher TMAO was associated with increased mortality, particularly in patients with eGFR < 90 mL/min/1.73 m^2^
Senthong V., 2016 [97]	Prospective cohort study	935 patients	Patients with peripheral artery disease (PAD) undergoing elective angiography	Measurement of plasma TMAO and 5-year follow-up for all-cause mortality	Higher TMAO independently predicted 5-year mortality across PAD patients and improved risk stratification beyond traditional factors
Roncal C., 2019 [98]	Prospective cohort study	262 patients	Patients with PAD	Measurement of plasma TMAO and ~4-year follow-up for cardiovascular mortality	Higher TMAO was associated with more severe PAD and independently predicted cardiovascular mortality
Roncal C., 2019 [99]	Prospective cohort study	262 patients	Patients with PAD	Measurement of plasma TMA, TMAO, and TMA/TMAO ratio with ~4-year follow-up for cardiovascular mortality	Higher TMAO, lower TMA/TMAO ratio, and higher TMA predicted PAD severity and cardiovascular death, combined TMA/TMAO ratio improved risk prediction beyond individual markers
Suzuki T., 2016 [100]	Prospective cohort study	972 patients	Patients with acute heart failure (AHF)	Measurement of plasma TMAO, follow-up for 1 year	Elevated TMAO predicted in-hospital and 1-year mortality, as well as death or HF rehospitalization. Prognostic value improved when combined with NT-proBNP but was attenuated after adjusting for renal function
Senthong V., 2016 [101]	Prospective cohort study	2235 patients	Patients with stable coronary artery disease who underwent elective coronary angiography	Measurement of plasma TMAO and 5-year follow-up	Higher TMAO predicted 5-year all-cause mortality independent of traditional risk factors, inflammation and renal function, and improved prognostic stratification.
Amrein M., 2022 [102]	Prospective cohort study	1726 patients	Patients with suspected fCAD	Measurement of TMAO, betaine, choline, carnitine and 5-year follow-up	TMAO and its precursors were higher in fCAD but had low diagnostic value, elevated TMAO predicted 5-year all-cause death and cardiovascular events independent of renal function

## Data Availability

No new data were created or analyzed in this study.

## Abbreviations

The following abbreviations are used in this manuscript:

AF	Atrial fibrillation
AHF	Acute heart failure
BCAA	Branched-chain amino acid
BNP	Brain natriuretic peptide
CKD	Chronic kidney disease
CRP	C-reactive protein
CVD	Cardiovascular disease
eGFR	Estimated glomerular filtration rate
fCAD	Functionally relevant coronary artery disease
FMO3	Flavin monooxygenase 3
HF	Heart failure
HFpEF	Heart failure with preserved ejection fraction
LV	Left ventricular
LVEF	Left ventricular ejection fraction
MACE	Major adverse cardiovascular events
NT-proBNP	N-terminal pro-B-type natriuretic peptide
PAD	Peripheral artery disease
RCT	Randomized controlled trial
ROS	Reactive oxygen species
T2D	Type 2 diabetes
TMA	Trimethylamine
TMAO	Trimethylamine N-oxide
